# Negative Pressure Wound Therapy for the Prevention of Surgical Site Infections Using Fascia Closure After EVAR—A Randomized Trial

**DOI:** 10.1007/s00268-022-06740-5

**Published:** 2022-10-14

**Authors:** Robert Svensson-Björk, Julien Hasselmann, Giuseppe Asciutto, Moncef Zarrouk, Jonas Björk, Linda Bilos, Artai Pirouzram, Stefan Acosta

**Affiliations:** 1 Vascular Diseases Research Unit, Department of Clinical Sciences, Lund University, Ruth Lundskogs gata 10, 205 02 Malmö, Sweden; 2 Vascular Center, Skane University Hospital, Malmö, Sweden; 3 Department of Vascular Surgery, Uppsala University Hospital, Uppsala, Sweden; 4 Division of Occupational and Environmental Medicine, Lund University, Lund, Sweden; 5 Clinical Studies Sweden, Lund University, Forum South, Lund, Sweden; 6 Vascular Surgery Unit, Department of Cardiothoracic and Vascular Surgery, Linköping University Hospital, Linköping, Sweden

## Abstract

**Background:**

Surgical site infections (SSI) in the groin after vascular surgery are common. The aim of the study was to evaluate the effect of negative pressure wound therapy (NPWT) on SSI incidence when applied on closed inguinal incisions after endovascular aneurysm repair (EVAR).

**Methods:**

A multicenter randomized controlled trial (RCT). Between November 2013 and December 2020, 377 incisions (336 bilateral and 41 unilateral) from elective EVAR procedures with the primary intent of fascia closure were randomized and included, receiving either NPWT or a standard dressing. In bilateral incisions, each incision randomly received the opposite dressing of the other side, thereby becoming each other’s control. The primary endpoint was SSI incidence at 90 days postoperatively, analyzed on an intention-to-treat basis. Uni and bilaterally operated incisions were analyzed separately, and their respective p-values combined using Fisher’s method for combining *P*-values. Study protocol (NCT01913132).

**Results:**

The SSI incidence at 90 days postoperatively in bilateral incisions was 1.8% (n = 3/168) in the NPWT and 4.8% (n = 8/168) in the standard dressing group, and in unilateral incisions 13.3% (n = 2/15) and 11.5% (n = 3/26), respectively (combined *p* = 0.49). In all SSIs, bacteria were isolated from incisional wound cultures. No additional SSIs were diagnosed between 90 days and 1 year follow-up.

**Conclusions:**

No evidence of difference in SSI incidence was seen in these low-risk inguinal incisions when comparing NPWT with standard dressings after EVAR with the primary intent of fascia closure.

**Clinical Trials:** NCT01913132.

**Supplementary Information:**

The online version contains supplementary material available at 10.1007/s00268-022-06740-5.

## Introduction

Surgical site infections (SSI) are a major concern after inguinal vascular surgery, increasing inpatient stay and patient morbidity [1]. It has also been reported that approximately 55% of all SSIs are preventable with the implementation of the current evidence-based strategies [2]. Decreasing SSI incidence is therefore highly prioritized.

Use of negative pressure wound therapy (NPWT), dressings with an active suction, on sutured incisions has for the last decade been proposed as a possible prevention of SSIs. The evidence on its efficacy has increased in recent years, with five meta-analyses of randomized controlled trials (RCTs) showing significant reductions in SSI incidence when using NPWT compared to standard dressings [3–7]. However, the included studies evaluated SSI incidence after mainly open inguinal revascularization procedures. The risk of developing an SSI differs greatly between different inguinal vascular surgical procedures, with incidences varying between approximately 2–5% in inguinal incisions after endovascular aneurysm repair (EVAR) [1, 8, 9] and 20–30% after open revascularization procedures [3]. To date, no study has evaluated NPWT dressings on incisions after EVAR procedures only. The aim of the present RCT was to evaluate if NPWT dressings on inguinal incisions after EVAR procedures decrease the risk of developing an SSI.

## Materials and methods

### INVIPS-trial

The INVIPS-trial was approved by the regional ethical review board in Lund (Diary number 2013/322 and 2016/886). A study protocol was registered a priori at clinicaltrials.gov (NCT01913132) [8]. The INVIPS-trial was reported in accordance with the CONSORT guidelines (CONSORT checklist in Supplemental Table 1).

### Population

All patients undergoing elective EVAR procedures between November 2013 and December 2020 at two vascular centers in Sweden, Skåne university hospital in Malmö and Örebro university hospital, were considered for study participation. Preoperative exclusion criteria were inability to understand the study instructions and purpose, age < 18 years old, inability to give informed consent or ongoing inguinal infection. Postoperative exclusion criteria were no inguinal incision (vascular hemostasis with percutaneous closure device [PCD] or non-inguinal arterial access); incorrect allocation of interventional or control wound dressings; withdrawn consent; re-operation with an inguinal incision for acute bleeding, peripheral ischemia, or stent graft-reintervention; or non-incisional related mortality within 90 days postoperatively. [8]

**Fig. 1:**
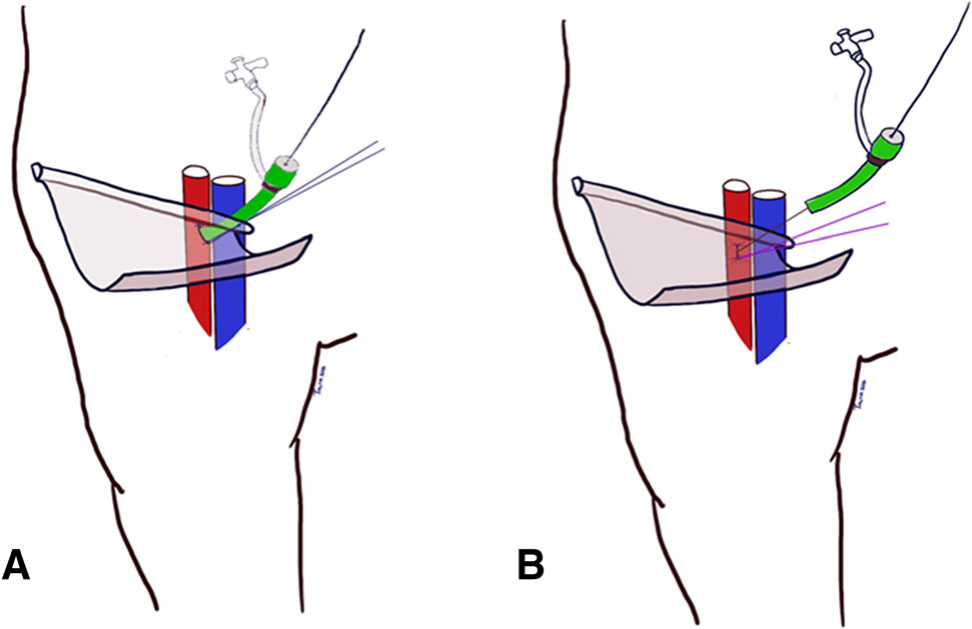
Schematic drawings illustrating the fascia closure suturing technique. A 4–6 cm long transversal skin incision is made over the access site. The fascia (curved translucent sheet) is exposed using blunt dissection. **a** A non-absorbable horizontal (lying) mattress suture (2–0 or larger) is placed in the fascia longitudinal to the artery (red) with the guidewire and introducer sheath and dilator in left in place (green). **b** The introducer is gently withdrawn along with tightening of the suture knot, while the guidewire acts as a safety wire. *Artist: Talha Butt*. If hemostasis is achieved and there is a palpable femoral pulse distal to the tied knot, the safety wire is removed. If there is a significant bleeding, the introducer with its dilator is reinserted over the safety wire to stop the bleeding and another horizontal mattress suture is placed close to the access site

All types of elective EVAR procedures including fenestrated EVAR and thoracic EVAR were included. The preoperative antibiotic prophylaxes used was the combination of 800 mg sulfamethoxazole and 160 mg trimethoprim orally, cloxacillin 2 g intravenously or clindamycin 600 mg intravenously in case of penicillin allergy. The preoperative skin-preparation, constituted of preoperative shower, hair removal with electric clipper if necessary, and antiseptic skin preparation using chlorhexidine gluconate. The surgical incisions in EVAR procedures were performed at the end of the endovascular treatment to achieve local hemostasis at the arterial access site. The primary technique for arterial access closure was bilateral fascia closure (Fig. 1) [10, 11]. As a bale-out procedure, if hemostasis was not achieved by fascia closure, cut-down with exposure of the common femoral artery for direct arterial suturing (abbreviated cut-down) was performed. Occasionally, the artery was exposed further for patch angioplasty where a patch of autologous, xenogenous or synthetic material was sutured over the arterial wall deficit.

Eligible patients were given written information about the study per ordinary mail and oral information at the outpatient clinic before written informed consent was retrieved from participating patients. Informed consent was retrieved before the surgical procedure was conducted.

### Randomization and wound dressing allocation

The enrolment of patients and the randomization process were conducted by nurses at the outpatient clinic, neither connected to the study investigators nor the operating surgeons. The random allocation sequence procedure was managed independently at the outpatient clinic by the draw of a randomization form from an opaque envelope prior to surgery. The randomization ratio was 1:1. Participating patients were randomized to receive either the NPWT dressing or the institutions’ standard dressing. In patients with bilateral incisions, the right inguinal incision was randomized while the contralateral incision received the opposite type of dressing. The NPWT dressing used was the PICO™ (Smith & Nephew, London, UK). The standard dressings used at the two centers during the seven-year study period were: ViTri Pad (ViTri medical, Stockholm, Sweden), Tegaderm + pad (3 M, Maplewood, Minnesota, US), Opsite post-op (Smith & Nephew, Hull, UK), or Mepilex boarder (Mölnlycke health care, Gothenburg, Sweden). Both the NPWT and standard dressings were applied under sterile conditions by the theater nurse while the patient was still at the operating theater. The NPWT dressings were changed if fully saturated with fluids or removed at 7 days postoperatively. The standard dressings were also changed if fully saturated with fluids or at the day of discharge.

### Outcomes

The primary endpoint of the present RCT was SSI incidence at 90 days postoperatively [8]. The secondary endpoints were SSI incidence at one year postoperatively and other wound complications (hematoma, wound dehiscence and seroma or lymphatic complications) at 90 days and one year postoperatively.

SSIs were defined using the predefined ASEPSIS-score [12] and criteria issued by Centers for Disease Control (CDC) [13] (Supplemental Tables 2 and 3). Identified SSIs were also graded according to the Szilagyi classification: grade 1 (involving dermis), grade 2 (involving subcutaneous tissue), or grade 3 (involving vascular prosthesis) [14]. The wound complications, both SSIs and other wound complications, were also graded according to an incisional wound complication scale [15].

The clinical wound manifestations were assessed by nurses and physicians independent from the conducted study during inpatient care and at the vascular outpatient clinic with a standardized follow-up visit 30 days postoperatively. A phone interview at 90 days postoperatively was conducted by the study nurse. Data from visits at primary care, emergency room and other surgical or vascular centers were collected retrospectively. Nurses and physicians were blinded to wound dressing allocations apart from during postoperative inpatient care.

Adverse events related to the NPWT dressing such as pain, skin blisters or technical problems were sought for and registered throughout the trial.

### Sample size

A sample size calculation was conducted a priori and has previously been published [8]. In the sample size calculation, the following assumptions were made: a reduction of SSI incidence through the use of NPWT dressing from 4.4 to 1%, a distribution of 80% bi and 20% unilateral incisions, a mortality rate at 1 year of 6.7%, an unspecified loss to follow-up of 10%, a statistical power of 80% and a significance level of 5%, resulting in an estimated demand of 497 incisions (398 bilateral and 99 unilateral). The enrolment of patients was terminated when the total number of randomized incisions was reached.

### Statistics

Data management and statistical analyses were conducted using SPSS software version 27 for Windows (IBM Corporation, New York, USA). The primary and secondary outcomes were analyzed on an intention-to-treat basis and presented per incision. The primary outcome was presented as frequencies and odds ratios (OR) with 95% confidence intervals (CI). Uni and bilateral incisions were analyzed separately. For frequencies of descriptive data, Fisher’s exact test was used in unilateral incisions while McNemar’s test was used in bilateral incisions. The obtained *p*-values of the separate analyses for unilateral and bilateral incisions were then combined using Fisher’s method of combining *p*-values [16]. *P*-values of < 0.05 were considered significant.

## Results

### Patients

**Fig. 2:**
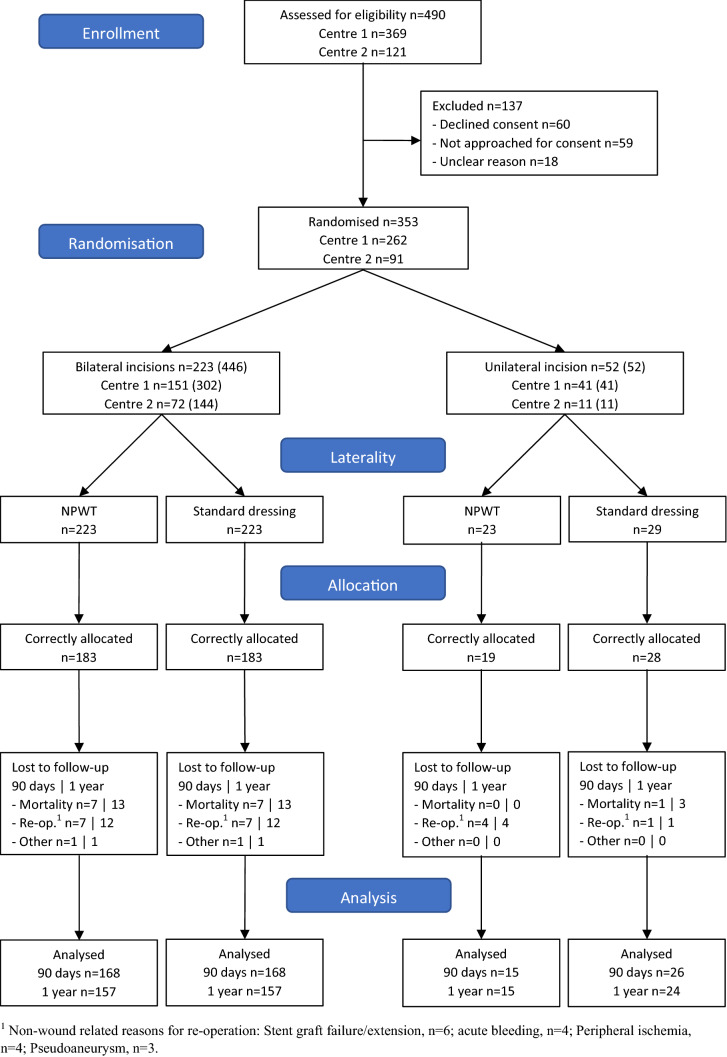
CONSORT flow diagram of INVIPS-trial EVAR-arm

Four hundred and ninety patients were assessed for eligibility of which 275 patients were randomized and received inguinal incisions (223 bilaterally and 52 unilaterally), resulting in 498 incisions. Of these, 66 patients (121 incisions) were excluded: 45 patients (85 incisions) due to not receiving the randomized wound dressing and 21 patients (36 incisions) were lost to follow-up. In total, 336 bilateral and 41 unilateral incisions were finally included in the analysis of the primary endpoint (Fig. 2).

The patient characteristics are listed in Table 1 and pre, peri and postoperative parameters are listed in Table 2. In patients operated bilaterally, the patient-related characteristics and parameters are identical and no differences among limb associated parameters were detected. In the unilaterally operated incisions, there was no difference in patient or pre, peri and postoperative parameters.

**Table 1:** Baseline characteristics of included patients

	Bilateral		Unilateral	
	Std dressing n = 168	NPWT n = 168	Std dressing n = 26	NPWT n = 15
Median age, years (IQR^1^)	73.3 (9.1)		74.3 (8.5)	75.2 (8.9)
Male sex (%)	146 (86.9)		18 (69.2)	10 (66.7)
Median BMI^2^, kg/m^2^ (IQR)	27.0 (5.1)		26.0 (5.8)	28.7 (6.9)
Hypertension (%)	130 (77.4)		21 (80.8)	15 (100.0)
Ischemic heart disease (%)	69 (41.1)		12 (46.2)	7 (46.7)
Peripheral artery disease (%)	9 (5.4)	9 (5.4)	6 (23.1)	0 (0.0)
Cerebrovascular disease (%)	26 (15.5)		0 (0.0)	4 (26.7)
Atrial fibrillation (%)	33 (19.6)		4 (15.4)	3 (20.0)
Diabetes mellitus (%)	34 (20.2)		2 (7.7)	5 (33.3)
Lifestyle treatment (%)	3 (1.8)		2 (7.7)	1 (6.7)
Non-insulin pharmacologic (%)	21 (12.5)		0 (0.0)	0 (0.0)
Insulin treatment (%)	10 (6.0)		0 (0.0)	4 (26.7)
Smoker				
Current (%)	42 (25.0)		4 (15.4)	2 (13.3)
Previous (%)	105 (62.5)		17 (65.4)	9 (60.0)
Previous vascular surgery (%)	22 (13.1)		9 (34.6)	6 (40.0)
Previous groin incisions (%)	23 (13.7)	27 (16.1)	9 (34.6)	6 (40.0)
Medication				
Anticoagulants (%)	32 (19.0)		5 (19.2)	4 (26.7)
Platelet inhibitor				
Single (%)	129 (76.8)		20 (76.9)	9 (60.0)
Dual (%)	7 (4.2)		1 (3.8)	1 (6.7)
Steroid treatment (%)	22 (13.1)		2 (7.7)	0 (0.0)
Ipsilateral foot wound (%)	2 (1.2)	1 (0.6)	0 (0.0)	0 (0.0)

^1^IQR, interquartile range

^2^BMI, body mass index

**Table 2:** Pre, peri and postoperative data of included patients

	Bilateral		Unilateral	
	Std dressing n = 168	NPWT n = 168	Std dressing n = 26	NPWT n = 15
*Preoperative*				
Anemia^1^ (%)	58 (34.5)		9 (34.6)	7 (46.7)
Antibiotic treatment (%)	2 (1.2)		0 (0.0)	0 (0.0)
Median albumin level, g/L (IQR^2^)	38.0 (5.0) n = 167		38.0 (4.0)	38.5 (7.5) n = 14
Median glucose level, mmol/L (IQR)	7.1 (2.9)		7.0 (2.0)	6.8 (5.8)
Median eGFR^3^, mL/min/1.73m^2^ (IQR)	71.0 (27.8)		63.0 (34.5)	60.0 (36.0)
*ASA*^*4*^* classification*				
Grade 2 (%)	20 (11.9)		4 (15.4)	1 (6.7)
Grade 3 (%)	134 (79.8)		19 (73.1)	10 (66.7)
Grade 4 (%)	14 (8.3)		3 (11.5)	4 (26.7)
*Perioperative*				
Antibiotic prophylaxis^5^ (%)	168 (100.0)		26 (100.0)	15 (100.0)
*Type of anesthesia*				
General (%)	165 (98.2)		24 (92.3)	14 (93.3)
Regional (%)	0 (0.0)		2 (7.7)	0 (0.0)
Local (%)	3 (1.8)		0 (0.0)	1 (6.7)
*Indication*				
Abdominal aortic aneurysm (%)	141 (83.9)		11 (42.3)	7 (46.7)
Thoracic aortic aneurysm (%)	10 (6.0)		7 (26.9)	3 (20.0)
Iliac aneurysm (%)	8 (4.8)		0 (0.0)	1 (6.7)
Endoleak (%)	8 (4.8)		2 (7.7)	1 (6.7)
Pseudoaneurysm (%)	1 (0.6)		4 (15.4)	0 (0.0)
Aortic dissection (%)	0 (0.0)		2 (7.7)	1 (6.7)
Stent migration (%)	0 (0.0)		0 (0.0)	2 (13.3)
*Type of surgery*				
EVAR^6^ (%)	115 (68.5)		8 (30.8)	6 (40.0)
Fenestrated EVAR (%)	34 (20.2)		5 (19.2)	2 (13.3)
Thoracic EVAR (%)	10 (6.0)		11 (42.3)	4 (26.7)
Redo-surgery (%)	9 (5.4)		2 (7.7)	3 (20.0)
Main device laterality (%)	83 (49.4)	85 (50.6)	18 (69.2)	11 (73.3)
*Type of arterial closure*				
Fascia closure (%)	120 (71.4)	117 (69.6)	18 (69.2)	8 (53.3)
Cut-down (%)	42 (25.0)	45 (26.8)	5 (19.2)	6 (40.0)
Patch angioplasty (%)	2 (1.2)	2 (1.2)	1 (3.8)	0 (0.0)
Patch angioplasty + adjunctive TEA^7^	1 (0.6)	2 (1.2)	1 (3.8)	0 (0.0)
Adjunctive TEA^7^ without patch (%)	0 (0.0)	0 (0.0)	0 (0.0)	1 (6.7)
Interposition graft (%)	1 (0.6)	0 (0.0)	1 (3.8)	0 (0.0)
Femoro-femoro crossover (%)	1 (0.6)	1 (0.6)	0 (0.0)	0 (0.0)
Unspecified (%)	1 (0.6)	1 (0.6)	0 (0.0)	0 (0.0)
*Skin closure*				
Intracutaneous sutures (%)	158 (94.0)	157 (93.4)	25 (96.2)	14 (93.3)
Percutaneous matrass (%)	1 (0.6)	2 (1.2)	0 (0.0)	0 (0.0)
Staples (%)	9 (5.4)	9 (5.4)	1 (3.8)	1 (6.7)
*Graft material at inguinal access site*				
Xenograft (%)	2 (1.2)	4 (2.4)	1 (3.8)	0 (0.0)
Synthetic graft (%)	3 (1.8)	1 (0.6)	1 (3.8)	0 (0.0)
Autologous graft (%)	0 (0.0)	0 (0.0)	1 (3.8)	0 (0.0)
Local hemostatic agent (%)	19 (11.3)	14 (8.3)	11 (42.3)	6 (40.0)
Median operation time, minutes (IQR)	193.5 (121.8)		201.0 (178.8)	180.0 (223.0)
*Postoperative*				
Intensive care (%)	26 (15.5)		11 (42.3)	6 (40.0)
Median in-hospital stay (IQR)	5.0 (2.0)		8.0 (6.5)	7.0 (6.0)
Prolonged antibiotic treatment (%)	10 (6.0)		3 (11.5)	1 (6.7)
> 2 units packed red blood cells (%)	26 (15.5)		7 (26.9)	4 (26.7)
Hyperglycemia^8^ (%)	42 (25.8) n = 163		11 (42.3)	4 (26.7)

^1^ Anemia, hemoglobin concentration of < 11.7 g/dL in females and < 13.4 g/dL in males

^2^ IQR, interquartile range

^3^ eGFR, estimated glomerular filtration rate

^4^ ASA, American society of anesthesiologists classification

^5^ Antibiotic prophylaxis, sulfamethoxazole 800 mg and trimethoprim 160 mg orally, cloxacillin 2 g intravenously or clindamycin 600 mg intravenously

^6^ EVAR, endovascular aneurysm repair

^7^ TEA, femoral thromboendarterectomy

^8^ Hyperglycemia, blood glucose concentration of > 200 mg/dL

### Outcomes

The primary outcome, SSI incidence at 90 days postoperatively, was in patients operated bilaterally 1.8% (n = 3/168 [Center 1, n = 3/111; Center 2, n = 0/57]) in the NPWT and 4.8% (n = 8/168 [Center 1, n = 6/111; Center 2, n = 2/57]) in the standard dressing group (*p* = 0.18; OR 0.29 [95% CI 0.03–1.50]) and in patients operated unilaterally 13.3% (n = 2/15 [Center 1, n = 0/9; Center 2, n = 2/6]) in the NPWT and 11.5% (n = 3/26 [Center 1, n = 2/24; Center 2, n = 1/2]) in the standard dressing group (*p* = 1.0; OR 1.02 [95% CI 0.80–1.30]), using both the ASEPSIS score or the CDC criteria to define SSIs (combined p-value of 0.49) (Table 3).

**Table 3:** Outcome data 90 days postoperatively

	Bilateral			Unilateral		
	Std dres n = 168	NPWT n = 168	p-value	Std dres n = 26	NPWT n = 15	*p*-value
*Surgical site infection (SSI)*						
ASEPSIS-score^1^ (%)	8 (4.8)	3 (1.8)	0.18	3 (11.5)	2 (13.3)	1.0
Satisfactory healing (%)	155 (92.3)	160 (95.2)		23 (88.5)	13 (86.7)	
Disturbed healing	5 (3.0)	5 (3.0)		0 (0.0)	0 (0.0)	
Minor SSI (%)	3 (1.8)	1 (0.6)		0 (0.0)	0 (0.0)	
Moderate SSI (%)	1 (0.6)	0 (0.0)		0 (0.0)	0 (0.0)	
Severe SSI (%)	4 (2.4)	2 (1.2)		3 (11.5)	2 (13.3)	
CDC^2^ criteria (%)	8 (4.8)	3 (1.8)	0.18	3 (11.5)	2 (13.3)	1.0
Superficial (%)	4 (2.4)	1 (0.6)		0 (0.0)	0 (0.0)	
Deep (%)	4 (2.4)	2 (1.2)		3 (11.5)	2 (13.3)	
Organ/space (%)	0 (0.0)	0 (0.0)		0 (0.0)	0 (0.0)	
*Szilagyi classification*						
1. Dermis (%)	4 (2.4)	1 (0.6)		0 (0.0)	0 (0.0)	
2. Subcutaneous (%)	4 (2.4)	2 (1.2)		3 (11.5)	2 (13.3)	
3. Prosthesis (%)	0 (0.0)	0 (0.0)		0 (0.0)	0 (0.0)	
Isolation of bacteria (%)	9 (5.4)	5 (3.0)		3 (11.5)	2 (13.3)	
Median time to SSI, days (IQR^3^)	14.0 (14.0)	8.0 (n/a)		7.0 (n/a)	18.0 (n/a)	
*Other wound complication*						
Hematoma (%)	15 (8.9)	16 (9.5)	1.0	5 (19.2)	3 (20.0)	1.0
Wound dehiscence (%)	6 (3.6)	4 (2.4)	0.73	3 (11.5)	2 (13.3)	1.0
Seroma/lymphatic complication (%)	8 (4.8)	3 (1.8)	0.29	1 (3.8)	0 (0.0)	1.0
*Clinical implication scale*						
Clinical implication scale > 1	11 (6.5)	4 (2.4)	0.065	4 (15.4)	2 (13.3)	1.0
1. Prolonged in-hospital stay (%)	0 (0.0)	1 (0.6)		0 (0.0)	0 (0.0)	
2. Extra outpatient visit (%)	6 (3.6)	1 (0.6)		1 (3.8)	0 (0.0)	
3. Readmission without surgery (%)	2 (1.2)	0 (0.0)		0 (0.0)	0 (0.0)	
4. Readmission with surgery (%)	3 (1.8)	2 (1.2)		3 (11.5)	2 (13.3)	
5. Wound related amputation (%)	0 (0.0)	0 (0.0)		0 (0.0)	0 (0.0)	
6. Wound related death (%)	0 (0.0)	0 (0.0)		0 (0.0)	0 (0.0)	
Pseudoaneurysm (%)	11 (6.5)	10 (6.0)		3 (11.5)	0 (0.0)	

^1^ ASEPSIS-score, Additional treatment, Serous discharge, Erythema, Purulent exudate, Separation of the deep tissues, Isolation of bacteria and duration of inpatient Stay. See Appendix supplementary Table 1

^2^ CDC, Centers for disease control and prevention

^3^ IQR, interquartile range

The secondary outcome, SSI incidence at one year postoperatively, was in patients operated bilaterally 1.9% (n = 3/157 [Center 1, n = 3/104; Center 2, n = 0/53]) in the NPWT and 4.5% (n = 7/157 [Center 1, n = 5/104; Center 2, n = 2/53]) in the standard dressing group (*p* = 0.29) and in patients operated unilaterally 13.3% (n = 2/15 [Center 1, n = 0/9; Center 2, n = 2/6]) in the NPWT and 12.5% (n = 3/24 [Center 1, n = 2/22; Center 2, n = 1/2]) in the standard dressing group (*p* = 1.0), using both the ASEPSIS score or the CDC criteria to define SSIs (combined *p*-value of 0.65).

All SSIs occurred within 90 days postoperatively, with no SSI diagnosed after 30 days (range 4–23 days). Most SSIs were diagnosed after hospital discharge, n = 10/16, 62,5% (bilateral: NPWT n = 1/3 [33.3%], Standard dressing n = 5/8 [62,5%]; unilateral: NPWT n = 2/2 [100.0%], Standard dressing n = 2/3 [66.7%]). In all SSIs, bacteria were isolated in microbiological analysis of incisional wound cultures (Supplemental Table 4). Sepsis was not reported following an SSI. One patient with bilateral incisions developed an aortic stent graft infection approximately 5 months postoperatively verified by PET-CT and microbiological culture from the aneurysm sac (*Enterococcus faecalis*). Both inguinal incisions healed without complications within 30 days postoperatively and the source of the aortic stent graft infection was never determined.

The other secondary outcomes, incidence of non-infectious wound complications (hematoma, wound dehiscence and seroma or lymphatic complications) showed no differences among incisions treated with NPWT compared to standard dressings (Table 3).

In bilaterally operated patients, 2.4% (n = 4/168) of the NPWT treated incisions and 6.5% (n = 11/168) of the standard dressing treated incisions (*p* = 0.065) received additional treatment according to the incisional wound complication scale. In unilaterally operated patients, 13.3% (n = 2/15) of the NPWT treated incisions and 15.4% (n = 4/26) of the standard dressing treated incisions (*p* = 1.0) needed additional treatment. No patient was amputated or died due to any incisional wound complication.

### Adverse events

Twelve of 183 (6.6%) patients receiving the NPWT reported adverse events. Nine did not tolerate the unit and tube (of which six had postoperative confusion), two experienced pain or discomfort and one was disturbed by the noise from the pump. Nine patients (5.4%) reported technical problems with the NPWT dressing, of which eight were leakage and one lack of suction. In eleven of 183 patients (6.0%), the NPWT treatment was discontinued prior to the recommended 7 days of treatment.

## Discussion

The present multicenter RCT showed no evidence of difference in SSI incidence in these low-risk inguinal incisions when comparing NPWT with standard dressings after EVAR with the primary intent of fascia closure. This finding does not support the routine use of NPWT dressings in uncomplicated inguinal incisions with fascia closure.

There was a trend toward fewer additional treatments due to any incisional wound complication in bilateral incisions treated with NPWT compared to standard dressings, despite no significant difference in incidence of SSI or other incisional wound complications. This highlights the importance to also report the clinical implication of incisional wound complications.

The number of incisions randomized (n = 498) met the predefined number published in the study protocol (n = 497). The proportion of bilateral incisions (89.6%) were higher than anticipated based on the study protocol (80.0%), increasing the statistical power. Despite the high number of incisions excluded due to incorrect allocation of wound dressings, the bilateral incisions included in analysis (n = 336) almost met the predefined number for bilateral incisions only (n = 340). The number of included unilateral incisions was lower than anticipated based on the power calculation, which is due to a lower proportion of patients operated unilaterally and a higher fraction of incorrectly allocated wound dressings. Nevertheless, combining the number of bilateral and unilateral incisions resulted in an adequately powered study.

The present multicenter RCT is the first to evaluate NPWT dressings on closed incisions after EVAR procedures only. Previous RCTs have not separated EVAR incisions from other inguinal vascular incisions, resulting in a much higher SSI incidence due to a higher SSI incidence in open inguinal revascularization procedures. The SSI incidence in the present RCT is considered similar to that of previous studies evaluating SSI incidence in incisions after EVAR procedures only [1, 8, 9]. This confirms the importance of separating incisions in EVAR procedures from open revascularization procedures when evaluating interventions to reduce SSI incidence.

Despite monitoring the patients for one year to capture low virulent prosthetic SSI (according to the CDC guidelines) [12], none were detected. One patient developed an aortic stent graft infection, however of unknown origin. The incidence of aortic stent graft infections in the present study was lower than the incidence of 1.4% previously reported in a retrospective study [17]. The low incidence of aortic stent graft infections could be due to a too short follow-up time since the reported median time to aortic stent graft infection in that study was 3.2 years. [17] All the SSIs in the present study were diagnosed within 30 days postoperatively, indicating no need for a prolonged wound surveillance time of up to 90 days.

In recent years the use of percutaneous closure devices (PCDs) instead of fascia closure or cut-down has increased. One systematic review with meta-analysis of observational studies has shown a significant decrease in SSI and seroma incidence but an increase in pseudoaneurysm incidence with PCD compared to cut-down technique [18]. In two systematic reviews with meta-analyses of RCTs only, no significant difference in SSI incidence was seen [19, 20]. In one of the reviews [20], a significant decrease in seroma/lymphorrhea incidence with PCD was demonstrated. Only one RCT has evaluated PCD compared to fascia closure, showing no difference in incidence of SSI and other incisional wound complications [11]. The exact extent of contemporary use of PCD compared to fascia closure and cut-down is to the authors unknown, but many centers (including present study centers) has begun using PCD, which appears to limit the implication of the results of the present RCT. The choice of arterial closure technique used should be a matter for the individual centers and operating surgeons to choose. The authors believe that there is still a role for fascia closure and cut-down technique despite the introduction of PCD into clinical practice since these open techniques remain as common rescue techniques when PCDs fail to achieve adequate hemostasis [21]. The success rate of PCD and fascia closure was in a systematic review of both RCTs and observational studies 63–100% for PCD and 87–99% for fascia closure. [22]

The results of the present multicenter RCT are intriguing but there are a few limitations to consider, such as that the standard dressing used varied by vascular center, and the fact that neither the patients nor treating hospital staff were blinded to allocated wound treatment during in-hospital care. However, the present study also has several strengths. First, the multicenter RCT study design increases generalizability of study results. Secondly, the inclusion and separate statistical analysis of both uni and bilaterally operated incisions, with bilateral incisions receiving both the NPWT and standard dressing, respectively, increases statistical power and minimizes potential confounding [23]. The high proportion of bilateral incisions (89.1%), which is higher than anticipated in the power calculation, adds further scientific strength to the results. Finally, the objectivity of the ASEPSIS score with 100% confirmation from microbiological cultures and diagnosis of SSI by staff at the outpatient clinic blinded to the study, adds objectivity to the otherwise subjective diagnosis of SSI.

## Conclusions

The SSI incidence after primary intent of fascia closure for EVAR procedures was low. The present multicenter RCT showed no evidence of difference in SSI incidence in these low-risk inguinal incisions when comparing NPWT with standard dressings after EVAR with the primary intent of fascia closure.

## Supplementary Information

Below is the link to the electronic supplementary material.

Supplementary file1 (DOCX 32 kb)
